# The Oxygen Evolution Reaction at MoS_2_ Edge Sites: The Role of a Solvent Environment in DFT-Based Molecular Simulations

**DOI:** 10.3390/molecules28135182

**Published:** 2023-07-03

**Authors:** Estefania German, Ralph Gebauer

**Affiliations:** 1 Department of Theoretical, Atomic and Optical Physics, University of Valladolid, 47011 Valladolid, Spain; estefania.german@uva.es; 2 The Abdus Salam International Centre for Theoretical Physics (ICTP), Strada Costiera 11, 34151 Trieste, Italy

**Keywords:** oxygen evolution reaction, water splitting, density functional theory, implicit solvent, MoS_2_, molybdenum disulfide

## Abstract

Density functional theory (DFT) calculations are employed to study the oxygen evolution reaction (OER) on the edges of stripes of monolayer molybdenum disulfide. Experimentally, this material has been shown to evolve oxygen, albeit with low efficiency. Previous DFT studies have traced this low catalytic performance to the unfavourable adsorption energies of some reaction intermediates on the MoS_2_ edge sites. In this work, we study the effects of the aqueous liquid surrounding the active sites. A computational approach is used, where the solvent is modeled as a continuous medium providing a dielectric embedding of the catalyst and the reaction intermediates. A description at this level of theory can have a profound impact on the studied reactions: the calculated overpotential for the OER is lowered from 1.15 eV to 0.77 eV. It is shown that such variations in the reaction energetics are linked to the polar nature of the adsorbed intermediates, which leads to changes in the calculated electronic charge density when surrounded by water. These results underline the necessity to computationally account for solvation effects, especially in aqueous environments and when highly polar intermediates are present.

## 1. Introduction

In the face of the urgent global need for sustainable and clean energy sources, solar and wind energy have emerged as viable alternatives to traditional fossil fuels. These renewable resources are not only abundant but also capable of significantly reducing the carbon footprint associated with energy production. However, a major challenge associated with these intermittent energy sources is their inability to provide a consistent supply of power in the absence of sunlight or wind. Hence, it is crucial to have effective energy storage solutions to harness the full potential of these renewable energy sources.

One promising approach to address this intermittency issue involves converting surplus energy from solar and wind sources into hydrogen via water splitting [1]. Hydrogen is a high-energy, non-polluting fuel that can be stored and transported with relative ease, offering a potential solution to energy storage and delivery [2]. Nevertheless, the water-splitting process, which involves separating water into hydrogen and oxygen, requires the presence of an efficient catalyst to be energetically feasible.

In the pursuit of viable catalysts for this purpose, a key focus has been the use of earth-abundant materials. Such materials are not only widely available, but they also circumvent the problems associated with the cost, rarity, and potential toxicity of more traditionally used precious-metal-based catalysts. As such, research has intensified to identify earth-abundant materials that can act as efficient catalysts for water-splitting.

Layered Transition-Metal Dichalcogenides (TMDs) have shown significant promise as catalysts in the water-splitting process [3,4,5]. These compounds, which are characterized by layers of transition metal atoms sandwiched between chalcogen atoms [6], have demonstrated unique catalytic properties, including favorable reaction kinetics and long-term stability. Moreover, their layered structure provides ample active sites for the reaction, which may further enhance their catalytic efficiency [7,8].

Among the layered TMDs, Molybdenum Disulfide (MoS_2_) is a prominent representative, which is attracting considerable attention in scientific research due to its exceptional properties and potential applications [3].

Apart from its emerging role as a catalyst in water-splitting, MoS_2_ has found many other applications in various fields. For instance, owing to its semi-conductive nature and impressive electron mobility, MoS_2_ has been utilized in the fabrication of transistors [9,10,11], making it a potential contender for next-generation electronics. Moreover, its unique photoelectric properties make it an ideal candidate for photovoltaic applications and optoelectronic devices [12]. The compound has also been used in the realm of energy storage, serving as an electrode material in lithium-ion batteries due to its high capacity for lithium intercalation [13]. Furthermore, the high surface area and strong adsorption capacity of MoS_2_ have led to its application in environmental fields, such as water- and air-purification technologies [14,15].

Recently, there has been particular interest in towards the catalytic properties of MoS_2_ monolayers, especially regarding the Oxygen Evolution Reaction (OER), a critical half-reaction in water-splitting. It is well-established that the edge sites of MoS_2_ are highly catalytically active [16,17]. For example, the HER is known to be catalysed by edges, not by the inert basal plane of MoS_2_ [18]. For the case of the OER, we have recently shown [19] that the pristine monolayer exhibits a strong over-binding of the O${}^{*}$ reaction intermediate, which leads to an unfavourable alignment of the energy levels [19]. Bulk MoS_2_ provides limited edge sites, restricting its overall catalytic performance. A monolayer of MoS_2_, with its two-dimensional structure, presents a significantly higher proportion of edge sites compared to its bulk counterpart. This maximization of active sites potentially enhances its catalytic efficiency, making monolayer MoS_2_ a promising catalyst for the OER. Indeed, layers of MoS_2_ and MoS_2_-WS_2_ heterojunctions have been experimentally observed to split water [20].

In the field of catalysis, theoretical and computational strategies are being increasingly recognized for their instrumental role in supplementing experimental observations [21]. These approaches not only provide a computational framework to validate empirical findings but also offer an interpretative lens to discern underlying catalytic mechanisms that are otherwise challenging to investigate experimentally. In this regard, Density Functional Theory (DFT) frequently emerges as the methodology of choice, due to its potency in calculating electronic structures and predicting chemical properties [22,23]. In the particular case of the OER, the key processes to understand are proton-coupled electron transfers (PCETs). The production of one molecule of O_2_ from water involves four distinct PCET steps. Even though these processes happen in a liquid environment, it is common practice to perform the corresponding DFT calculations in a vacuum, i.e., in a setup where the reaction intermediates are bonded to the catalyst surface, but the (water) environment surrounding the catalyst and reactants is neglected.

Approximate methods to account for the presence of a liquid environment have existed for many years. They are typically focused on mimicking the effects of the dipolar nature of polar solvents such as water. Most commonly, one resorts to so-called continuum solvation models where a dielectric is defined as a smooth self-consistent function of the electronic density of the solute. Such methods have very successfully been applied in the realm of theoretical chemistry, while applications in condensed matter or solid-state physics are more rare.

In this paper, we apply the revised self-consistent continuum solvation (SCCS) methodology to the edges of monolayer MoS_2_. We show how the strong dipoles present in typical OER reaction intermediates lead to important corrections of the adsorption energies and hence to a non-negligible correction of the estimated overpotential for the OER in this system.

## 2. Methodologies

### 2.1. The Implicit Solvent Model

Implicit solvent models, also known as continuum models, have been an integral part of computational chemistry and molecular simulations for several decades [24,25,26]. These models aim to describe the solvation environment around a molecule by approximating the solvent as a continuous medium, thereby bypassing the need to explicitly represent each solvent molecule. This simplification significantly reduces the computational demand and enables the study of larger systems or longer timescales than can typically be achieved with explicit solvent models. Many different continuum solvation models have been developed, predominantly within the realm of chemistry, since Onsager’s seminal contributions [27]. Among the multitude of such methodologies, one of the most well-received is the Polarizable Continuum Model (PCM) advanced by Tomasi and his colleagues [25,26]. The latest iteration of PCM incorporates a broad spectrum of similar techniques, such as the Conductor-like Screening Model (COSMO) approach [24]. Despite its popularity, PCM’s utilization has largely been confined to the chemistry discipline, and it remains relatively unexplored within the spheres of condensed matter and solid-state simulations. Specifically, the capacity to handle metallic systems within the PCM framework was subsequently introduced in an implicit manner [28]. Moreover, the corresponding algorithm was not inherently structured to interface with periodic systems and ab initio molecular dynamics (MD) simulations, further limiting its application in these domains.

Here, we employ an implicit solvation methodology that was first proposed by Fattebert and Gygi [29,30] and is well-suited to plane-wave pseudopotential calculations in periodic systems. In this approach, the dielectric function is defined as a smooth self-consistent function of the electronic density of the solute. A modified version of this procedure was suggested by Andreussi, Darbi and Marzari [31]. It overcomes several of the limitations of previous approaches. Their methodology is publicly available in the quantum-ENVIRON software, which is interfaced with, among others, the  quantum-ESPRESSO suite of programs [32].

This methodology, which is also employed here, involves defining an interface function, s(r), with respect to certain degrees of freedom within the system. The design of this interface function is such that it demonstrates a smooth transition from a value of 1 within the volume where the system’s degrees of freedom exist to a value of 0 in the region of embedding. This progression ensures a comprehensive representation of the embedded system while also capturing the nuances of its interaction with the embedding environment. In the case of SCCS, which are used here, the interface function is defined in terms of the solute’s electron density ρ(r) in the following way:

(1) $$s\left(r\right)=\left\{\begin{array}{cc} 0 & \rho \left(r\right)\leq \rho_{min}\\ 1-t\left(\frac{\log\left(\rho_{max}\right)-\log\left(\rho \left(r\right)\right)}{\log\left(\rho_{max}\right)-\log\left(\rho_{min}\right)}\right) & \rho_{min}<\rho \left(r\right)<\rho_{max}\\ 1 & \rho \left(r\right)\geq \rho_{max}, \end{array}\right.$$

where t(x) is a smooth step function going from 0 to 1 and is defined as $t\left(x\right)=x-\sin\left(2\pi x\right)/2\pi$.

The limiting values $\rho_{min}$ and $\rho_{max}$ determine how close or distant the surface s(r) is from the solute. In terms of the interface function s(r), the dielectric function ϵ(r) is defined as

(2) $$\epsilon \left(r\right)=\exp\left(\log\epsilon_{0}\cdot \left(1-s(r)\right)\right).$$

Once the dielectric function is defined as a function of s(r), the electrostatic potential ϕ(r) of the system in the presence of the continuum solvent is given by a generalized Poisson equation:

(3) ∇·ϵ(r)∇ϕ(r)=−4πρ(r).

The modified electrostatic potential ϕ(r) stabilizes the electronic densities of localized charges, and thus provides an electrostatic stabilization, especially in charged or dipolar systems like those present in the OER. The implicit solvent, which represents the effect of polar water molecules, can, in this way, play an important role in the stabilization of various reaction intermediates.

### 2.2. Model System of the MoS_2_ Edge

Molybdenum disulfide (MoS_2_), analogously to most TMDs from groups 4–7, exhibits a graphite-analogous layered lattice structure. Each stratified unit of this structure comprises a triadic arrangement of atom planes: a central plane of molybdenum (Mo) atoms lies between two exterior planes of sulfur (S) atoms. The spatial separation of these atomic planes is approximately 3.14 Å.

Intralayer bonding within this structure predominantly involves covalent interactions between the Mo and S atoms in a hexagonal lattice. This lattice exhibits a trigonal prismatic ($D_{3h}$ point group) configuration, frequently designated as 2H [33,34].

**Figure 1:**
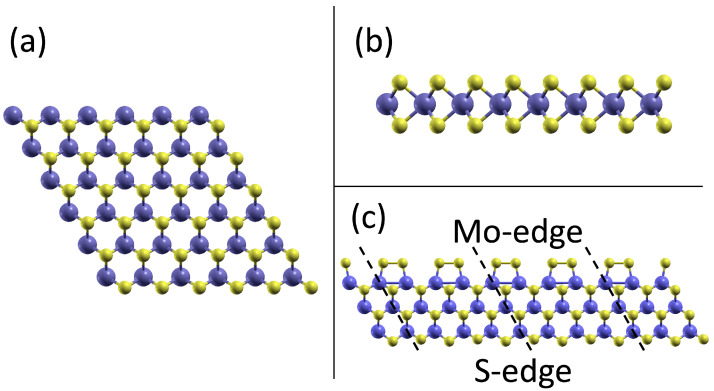
Structure of the MoS_2_ model systems. (**a**) Pristine MoS_2_ monolayer, top view. (**b**) Side view of a pristine monolayer. (**c**) Infinite stripe model in top view. The two inequivalent edges (sulfur edge and molybdenum edge, respectively) can be discerned. See the main text for a description of the edges. The supercell along the axis of the stripe is indicated by the dashed lines. Colour code: Sulfur (yellow) and molybdenum (dark blue).

In macroscopic manifestations of MoS_2_, these layers are bonded primarily through weak, non-directional van der Waals (vdW) forces. This form of interaction underlies the facile cleavage of the bulk material into individual monolayers. Figure 1a,b graphically depicts such a representative monolayer of MoS_2_ [35,36].

As we are interested in the catalytic properties of a single monolayer, we model this within periodic boundary conditions by including about 15 Å  of vacuum between repeated images of the layer. This distance is enough to effectively decouple adjacent layers.

The edge of the layer is represented by an *infinite stripe model*. A 4×4 MoS_2_ supercell is periodically repeated in one direction only, which leads to a stripe that exposes two inequivalent edges; see Figure 1c. One edge, known as the *sulfur edge*, exposes the outermost sulfur atoms of the hexagonal lattice. As it is the case inside the MoS_2_ layer, there are two sufur atoms at the edge, one above and one below the Mo layer. Each S-atom is bonded to two of the outermost Mo atoms. The sulfur edge is therefore obtained from a simple cut of the pristine MoS_2_ layer.

The second kind of edge is called *molybdenum edge*. It is created by a cut from the pristine layer with Mo atoms at the edge, followed by decorating the edge with one sulfur atom per Mo atom. In the minimum energy configuration of this edge, the terminating S atoms form pairwise bonds with one of their edge neighbours, with a long bonding distance of 2.19 Å. The molybdenum edges are thus terminated by pairs of weakly bound sulfur atoms, as shown in Figure 1c.

Such models for the edges of MoS_2_ layers have frequently been studied in the context of catalysis. In Ref. [37], the stability and structures of the considered Mo and S edges are examined by DFT. In Ref. [38], DFT calculations and experiments are used to study the role of transition metal atoms on the catalytic activities of the edges. Very recently, a joint experimental and computational study of MoS_2_ edges highlighted the role of noble metals at such edges [39].

### 2.3. Water Splitting and PCETs

The splitting of water into oxygen and hydrogen,

(4) $$2H_{2}O⟶2H_{2}+O_{2},$$

can be written as two separate half-reactions:

(5) $$2H^{+}+2e^{-}⟶H^{2},$$

known as the hydrogen evolution reaction (HER) and

(6) $$2H_{2}O⟶4H^{+}+4e^{-}+O_{2},$$

which is the oxygen evolution reaction (OER). As  is clear from Equation (6), the OER involves four distinct deprotonations, each one coupled with the transfer of an electron, i.e., four PCETs. In order to assess the energy needed for the OER in Equation (6), one should have access to the (free) energy of a proton and an electron, as they appear on the right-hand side of Equation (6). It is very difficult to evaluate those two energies separately, but one can use the fact that the relevant quantity is the *sum* of both energies. Using the fact that the free energy of (H^+^ + e^−^) is equal to half the energy of a hydrogen molecule under standard conditions, it is possible to avoid the calculation of a proton’s or an electron’s energy by replacing their sum with $\frac{1}{2}E(H_{2})$. This is known as the “computational hydrogen electrode” (CHE), and was first used by Nørskow and collaborators [40].

The CHE only provides the energy difference between subsequent reaction intermediates, with one difference for each PCET. It does not provide kinetic information, for which the barriers between the initial and final state would also need to be known.

One particularly important barrier is the formation of the O−O bond. This formation is non-trivial because of the spin polarization of the oxygen molecule [41]. As a direct simulation of this step goes beyond the CHE approach used here; we point the interested reader to a recent review about modeling of elementary steps in electrocatalysis [42]. Work along the lines described in Refs. [42,43] is currently under way to study the O−O bond formation at MoS_2_ edges.

**Figure 2:**
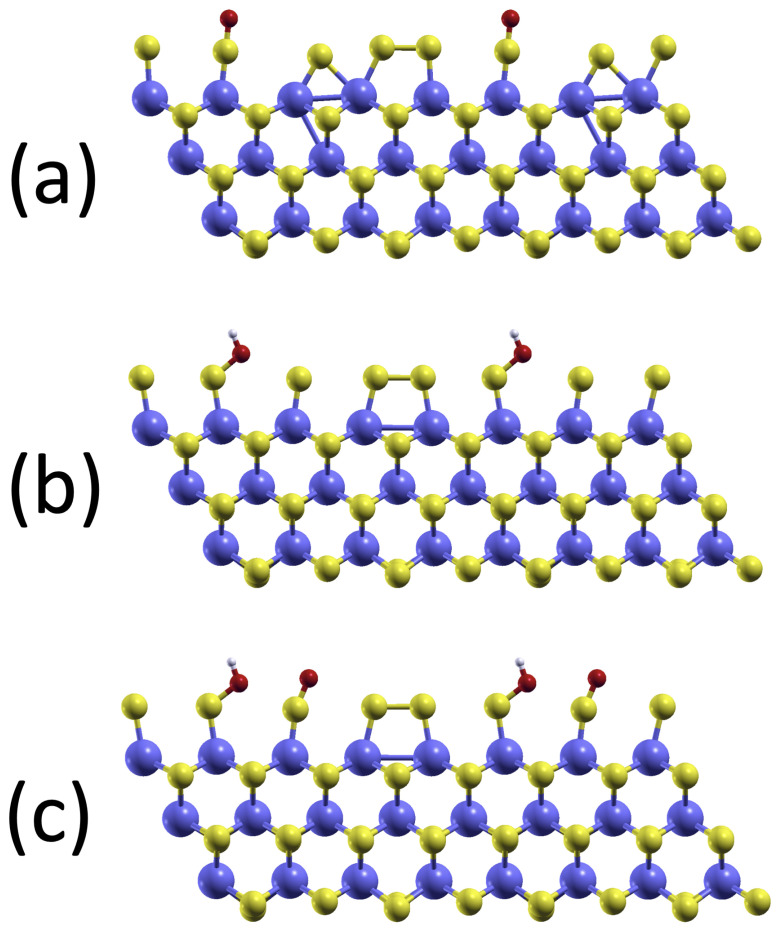
Infinite stripe model of MoS_2_ with key reaction intermediates: (**a**) Oxygen O${}^{*}$. (**b**) Hydroxyl group HO${}^{*}$. (**c**) HOO${}^{*}$, which binds in the lowest energy configuration as HO${}^{*}$ + O${}^{*}$. Colour code: Sulfur (yellow), molybdenum (dark blue), oxygen (red), hydrogen (grey).

Here, we consider the following four PCETs, which are established in the OER:

(7) $$\begin{array}{ccc} 2H_{2}O+{\left(\right)}^{*} & \overset{A}{⟶} & HO^{*}+H_{2}O+\left(H^{+}+e^{-}\right) \end{array}$$

(8) $$\begin{array}{ccc} & \;\;\;\;\;\;\;\;\;\;\;\;\;\;\;\;\;\;\;\overset{B}{⟶} & O^{*}+H_{2}O+2\left(H^{+}+e^{-}\right) \end{array}$$

(9) $$\begin{array}{ccc} & \;\;\;\;\;\;\;\;\;\;\;\;\;\overset{C}{⟶} & HOO^{*}+3\left(H^{+}+e^{-}\right) \end{array}$$

(10) $$\begin{array}{ccc} & \;\;\;\;\;\;\;\;\;\;\;\;\;\;\;\;\;\;\;\overset{D}{⟶} & {\left(\right)}^{*}+O_{2}+4\left(H^{+}+e^{-}\right), \end{array}$$

where the *-symbol indicates binding to the catalyst. From steps *A* to *D* above, it can be seen that, apart from the pristine MoS_2_ edge, three more systems with reaction intermediates bound to the edge are needed, namely O${}^{*}$, HO${}^{*}$, and O*HO${}^{*}$. The MoS_2_ infinite stripe with the reaction intermediates is shown in Figure 2a–c in their minimum energy configuration.

In order to evaluate the energetics of the four PCET steps above, the relevant quantity is the Gibbs free energy. Apart from the internal energy, calculated as the DFT total energy, also the pressure-volume work and entropic contributions are part of the Gibbs free energy. By far the largest corrections to the internal energy are linked to the free energy of the gas-phase O_2_ and H_2_ molecules. In this work, we follow the usual practice [44] to neglect entropic contributions to the reaction intermediates, which are bound to the catalyst, and consider the gas-phase molecules at standard conditions.

### 2.4. Density Functional Theory Calculations

Using spin-polarized Density Functional Theory (DFT), all computations are carried out with the quantum-ESPRESSO software, a comprehensive suite designed for the exploration and post-processing of electronic structures and molecular and condensed systems. The exchange and correlation functional is approximated with the semi-local gradient-corrected form, as parametrized by Perdew, Burke, and Ernzerhof (PBE). We use a plane-wave basis set throughout. The kinetic energy cutoffs for the orbitals and the charge density have been set to 45 Ry and 360 Ry, respectively. It was carefully verified that these cutoffs lead to well-converged energies and forces. The interaction between electrons and ions is modeled by employing Vanderbilt’s ultrasoft pseudopotentials (USPP). Concretely, we use the pseudopotentials Mo_ONCV_PBE-1.0.oncvpsp.upf (valence charge $Z_{val}=14$), s_pbe_v1.4.uspp.F.UPF ($Z_{val}=6$), O.pbe-n-kjpaw_psl.0.1.UPF ($Z_{val}=6$), and H.pbe-rrkjus_psl.1.0.0.UPF ($Z_{val}=1$) for molybdenum, sulfur, oxygen, and hydrogen, respectively. These pseudopotentials can be found in the Standard Solid-State Pseudopotentials (SSSP) database [45,46].

The Brillouin zone (BZ) of this quasi-one-dimensional edge system is sampled using a 4×1×1 regular grid of *k*-points with a shift [47]. For the occupation numbers of the KS orbitals, we use the “cold” smearing method of Marzari [48] with an electronic temperature of 136 meV.

In order to account for the weak dispersion interactions, which can play an impotant role in weak binding regimes, we also include the correction known as Grimme-D3 [49].

## 3. Results and Discussion

### 3.1. Electronic and Magnetic Structure

**Figure 3:**
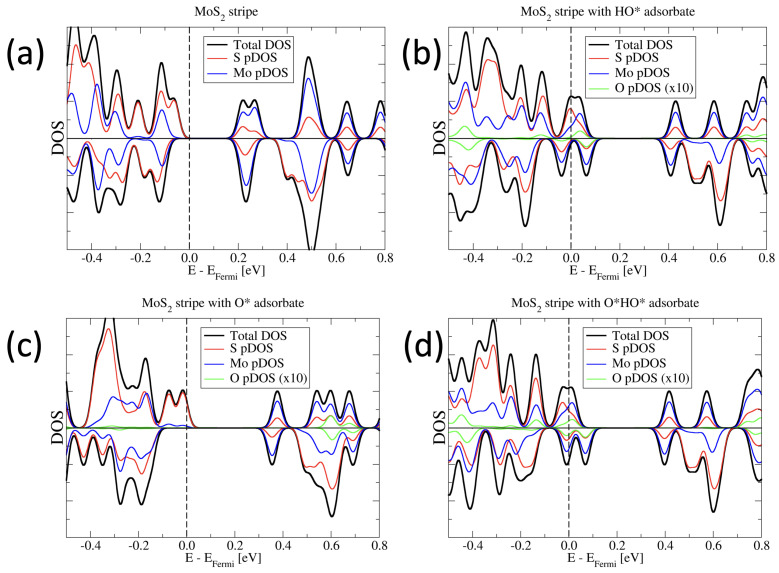
Spin resolved density of states (DOS) and projected DOS (pDOS) of the systems considered. The spin-up and spin-down DOS is shown as positive and negative DOS, respectively. (**a**) Pristine MoS_2_ stripe without adsorbates. (**b**) Stripe with HO${}^{*}$ adsorbate. (**c**) Stripe with O${}^{*}$ adsorbate. (**d**) Stripe with O*HO${}^{*}$ adsorbate. The pDOS of the adsorbed oxygen atoms is multiplied by a factor of 10 for better visual clarity.

In a first step, we consider the density of states (DOS) for the MoS_2_ stripes with and without the reaction intermediates adsorbed. In Figure 3a, we depict the pristine stripe. It is characterized by a band gap of 0.28 eV. The valence band edge is dominated by majority-spin (spin-up) states that are mainly localized on sulfur atoms. The conduction band edge, in contrast, is dominated by Mo states. Clearly, the main exchange splitting in the spin channels can be observed for the sufur states. This is in agreement with the observation of the main magnetic polarization, which is localised on S atoms at the sufur edges of the stripe.

When the reaction intermediates are bonded to the stripe, it loses its semi-conducting nature. In all cases, the majority spin channel is characterized by a partially occupied sulfur-state close to the Fermi level. In the cases of the HO${}^{*}$ and O*HO${}^{*}$ adsorbates, the minority spin channel features a partially occupied state, mostly of Mo character. The case of the O${}^{*}$ adsorbate is semi-metallic, with a band gap of 0.4 eV in the minority spin channel.

Interestingly, the adsorbate atoms themselves, i.e., the oxygen atoms, contribute to the DOS close to the Fermi energy only in the cases of the HO${}^{*}$ and O*HO${}^{*}$ adsorbates, but not when a single oxygen atom is adsorbed on the stripe.

In none of the considered cases is any magnetic moment localized on the adsorbed atoms of the reaction intermediates. Instead, the magnetization is localized at the sulfur edge of the stripe.

### 3.2. Energetics of the OER

In order to evaluate the effect of the liquid solvent on the OER, we consider the particular case of the edge of a MoS_2_ stripe as catalytic site. Using the common practice of considering catalyst and adsorbed reaction intermediates in a vacuum environment, it has previously been shown that the molybdenum edge is the more advantageous site for the OER on MoS_2_, with an overpotential of 1.15 eV [19]. In the following, we will study the OER at such Mo-edges when the water environment is taken into account and compare the key results with the standard computations in vacuum.

The geometry of Mo-edges in the minimum energy configuration is unaltered by the inclusion of an implicit solvent model: weakly bonded pairs of sulfur atoms decorate the molybdenum atoms at the edge, as shown in Figure 1c above. When it comes to adsorbing the three reaction intermediates, their bonding geometries are shown in Figure 2, and the corresponding bond lengths are summarized in Table 1. The general picture of the adsorption remains unaltered by the inclusion of the solvent: one of the two pairs of edging sulfur atoms is broken, and the intermediates bind to one (for the case of O${}^{*}$ and HO${}^{*}$) or both (for the case of O*HO${}^{*}$) of these sulfur atoms.

Looking at Table 1, it is notable that the bonding distances are little affected by the presence of the water solvent. In fact, the bond lengths between the edging sulfur and the O${}^{*}$ adsorbate are essentially the same in both cases. The same is true for the O−H bond, both in the HO${}^{*}$ adsorbate and in the O*HO${}^{*}$ cases, where the bond length varies by less than 1 percent. The only significant change in bonding is observed for the bond length of the edging sulfur atom with the oxygen of the HO${}^{*}$ group, both for the case of the HO${}^{*}$ and the O*HO${}^{*}$ intermediates. In this case, the inclusion of the water solvent results in a reduction in the bond length of about 2.5%.

**Figure 4:**
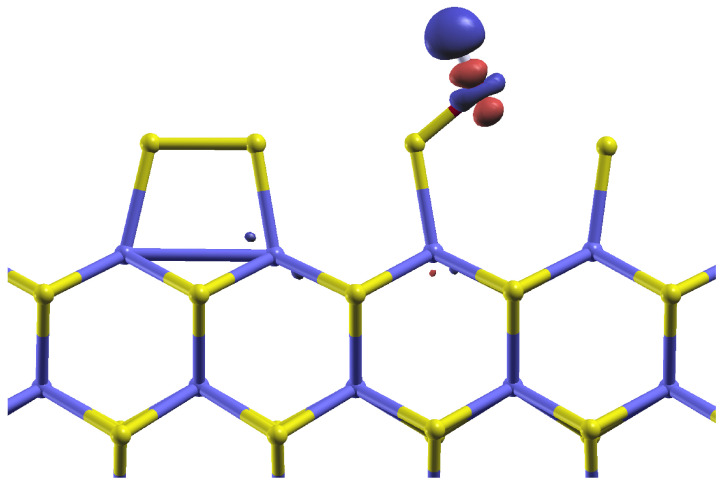
Reaction intermediate HO${}^{*}$ adsorbed on the molybdenum edge of the MoS_2_ stripe. A contour plot of the charge density difference between the solvated system and the system in vacuum is presented. The geometry is that of the solvated system. The blue-coloured isosurface corresponds to a reduction in the charge density in the solvated system, while the red colour corresponds to an increase in charge density due to solvation.

In order to understand the influence of the water solvent on the bonding of the hydroxyl group, we plot, in Figure 4, the difference in charge density $\rho_{water}-\rho_{vacuum}$ for the case of the HO${}^{*}$ intermediate, where $\rho_{water}$ is the electronic charge density obtained with the implicit solvent model and $\rho_{vacuum}$ the charge density without any solvent. Both charge densities were calculated in the same geometry, namely that of the system relaxed with water solvent. Clearly, the presence of the (polar) solvent leads to a reduction in the electronic charge around the proton and an increase around the oxygen adatom, with the shape of a d-orbital. It is this population of the polarization orbital on O that goes along with the reduction in the bonding distance to the edging sulfur atom. In Figure 4, the only places at which where there is a significant change in electronic density due to the solvent are around the adsorbed hydroxyl group.

Considering only the adsorption geometry, the influence of the solvent thus seems negligible. This picture changes when the adsorption energies are considered. We define the adsorption energies $E_{ads}$ of the various intermediates in the following way:

(11) $$E_{ads}=E_{MoS_{2}+\mathrm{intermediate}}-E_{MoS_{2}}-N_{O}\mu_{O}-N_{H}\;\mu_{H},$$

where $E_{MoS_{2}+\mathrm{intermediate}}$ is the DFT energy of the MoS_2_ edge system with the reaction intermediate, as shown in Figure 2a–c. $E_{MoS_{2}}$ is the DFT energy of the pristine MoS_2_ stripe of Figure 1c. $N_{O}$ and $N_{H}$ are the numbers of oxygen and hydrogen atoms in the considered intermediate, respectively. The chemical potentials $\mu_{O}$ and $\mu_{H}$ of oxygen and hydrogen are obtained as half the energies of O_2_ and H_2_ molecules. For the systems considered in water or vacuum, all energies and chemical potentials appearing in Equation (11) are calculated with or without implicit solvent contributions, as detailed in Section 2.1.

The resulting adsorption energies are summarized in Table 2. The presence of the water environment strongly reduces the adsorption strength of all considered reaction intermediates. The strongest reduction is observed for the O*HO${}^{*}$ intermediate, where the solvent leads to a reduction in the adsorption energy of 0.37 eV.

Linked to the adsorption energies of the intermediates are the energies involved in the four PCET steps A–D from Equations (7)–(10). The energy required for each step is defined as the energy difference between the products and reactants. In Table 3, we report the height of each one of these steps, both for the case of vacuum (from Ref. [19]) and water solvation (this work). The over-potential of the overall OER reactions is given by the difference between the highest step (step D in both cases considered here) and the ideal step height of 1.23 eV.

The data from Ref. [19] in Table 3 show that the molybdenum edge of MoS_2_ stripes has an overpotential of 1.15 eV for the OER, which seriously reduces the efficiency of this catalyst. This overpotential can be traced back to the too-strong binding of all the reaction intermediates to the stripe, which results in the huge overpotential for the PCET step *D* in Equation (10). It is therefore clear that the reduction in adsorption energy due to the solvent can play a role in reducing the overpotential.

**Figure 5:**
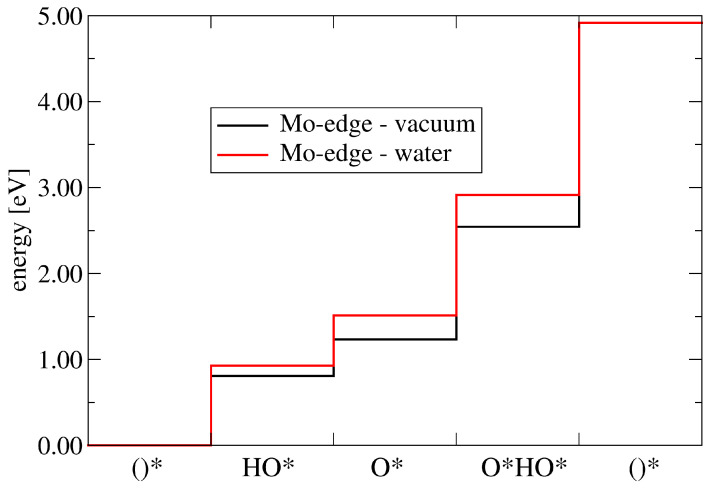
Energy diagram for the four PCET steps of the OER, Equations (7)–(10). The step heights are strongly dependent on the solvation of the catalyst.

Figure 5 shows the energy-level diagram for the four PCET steps, calculated using the methodology summarized in Section 2.3, corresponding to the data in Table 3. The reduction in the adsorption energy leads to the increased step heights of the PCET steps *A* to *C* (Equations (7)–(9)), and hence to a net reduction in the overpotential determining step *D*. Considering the water solvent, PCET step *D* has now a height of 2.0 eV, compared with 2.38 eV in the vacuum case. The calculated reaction overpotential is therefore reduced to 0.77 eV by the introduction of the implicit solvent. This difference shows how important it is to properly account for the liquid environment in which the OER takes place. Calculations in vacuum lead to similar adsorption structures, but the relative energies of the reaction intermediates may change considerably, as is the case in this system.

## 4. Conclusions

We performed DFT-based computer simulations of the OER at Mo-edges of MoS_2_ sheets. This TMD is intensely studied in the context of various applications in catalysis, and experimentally shown to be able to evolve O_2_ [20], but with low efficiency. In a previous DFT study [19], the binding strength of some of the reaction intermediates was found to be unfavorable for the OER, leading to the observed large overpotential and low activity for the O_2_ evolution.

These previous DFT simulations were based on a computational model where the catalyst and the adsorbed reaction intermediates are placed in a vacuum, at variance with the experimental setup, where the catalyst is in aqueous solution. The key point of the present work is to go beyond this common approximation and estimate the effect of a water solvent surrounding the MoS_2_ edges. This is achieved by applying an implicit solvent model, which can account for some of the main effects that the solvent has on the system by embedding the system in a realistic dielectric medium.

Our calculations, which include such solvent effects, show that the adsorption geometries and reaction mechanics of the OER are only very weakly altered by the presence of the solvent. Comparing the system’s electronic charge density with and without the dielectric medium reveals that the main effect of the solvent is localized in space, close to the adsorbed reaction intermediates. These changes in the charge density, and hence in the electrostatic interactions, have a strong effect on the energies of adsorption. In the case of the Mo-edges of MoS_2_ sheets studied here, the variance in adsorption energies leads to a reduction in the OER’s overpotential from 1.15 eV to 0.77 eV. This considerable change in the overpotential exemplifies that DFT-based computer simulations of reactions in liquid environments should study solvation effects whenever possible.

In a future study, we plan to extend the current computational approach by going beyond a continuum solvent model and explicitly including water molecules. Such an approach [50], which combines molecular dynamics simulations with the continuum models employed here, will allow for an even more detailed view of the energetics and reaction mechanics of the OER in a real application.

## Figures and Tables

**Table 1:** Adsorption geometries of reaction intermediates at the MoS_2_ molybdenum edge with and without solvation in water. Distances in Å.

Solvation	Intermediate	d(S−O)	d(O−H)	d(S−O(H))
	O*HO${}^{*}$	1.481	0.979	1.704
Vacuum	HO${}^{*}$	-	0.980	1.702
	O${}^{*}$	1.480	-	-
	O*HO${}^{*}$	1.485	0.986	1.661
Water	HO${}^{*}$	-	0.986	1.661
	O${}^{*}$	1.482	-	-

**Table 2:** Adsorption energies of reaction intermediates at the MoS_2_ molybdenum edge with and without solvation in water. Energies in eV.

Solvation	Intermediate	$E_{\mathrm{ads}}$
	O*HO${}^{*}$	−2.375
Vacuum	HO${}^{*}$	−1.651
	O${}^{*}$	−1.225
	O*HO${}^{*}$	−2.003
Water	HO${}^{*}$	−1.530
	O${}^{*}$	−0.946

**Table 3:** Step heights for PCET steps A–D, Equations (7)–(10). Energies in eV.

Solvation	PCET Step	ΔE
	A	0.809
Vacuum [19]	B	0.426
	C	1.311
	D	2.375
	A	0.929
Water	B	0.585
	C	1.403
	D	2.004

## Data Availability

Not applicable.
